# Challenges in Assembling the Dated Tree of Life

**DOI:** 10.1093/gbe/evae229

**Published:** 2024-10-30

**Authors:** Carlos G Schrago, Beatriz Mello

**Affiliations:** Department of Genetics, Federal University of Rio de Janeiro, Rio de Janeiro, Brazil; Department of Genetics, Federal University of Rio de Janeiro, Rio de Janeiro, Brazil

**Keywords:** phylogenomics, horizontal gene transfer, multispecies coalescent, timescales, networks, reticulation

## Abstract

The assembly of a comprehensive and dated Tree of Life (ToL) remains one of the most formidable challenges in evolutionary biology. The complexity of life's history, involving both vertical and horizontal transmission of genetic information, defies its representation by a simple bifurcating phylogeny. With the advent of genome and metagenome sequencing, vast amounts of data have become available. However, employing this information for phylogeny and divergence time inference has introduced significant theoretical and computational hurdles. This perspective addresses some key methodological challenges in assembling the dated ToL, namely, the identification and classification of homologous genes, accounting for gene tree-species tree mismatch due to population-level processes along with duplication, loss, and horizontal gene transfer, and the accurate dating of evolutionary events. Ultimately, the success of this endeavor requires new approaches that integrate knowledge databases with optimized phylogenetic algorithms capable of managing complex evolutionary models.

**Significance**

Assembling a dated phylogeny of life is essential for understanding Earth's biodiversity and its evolutionary history. However, traditional models of vertical inheritance of genes are inadequate to fully describe the intricacies of the evolution of species. Advances in genome sequencing and computational phylogenetics offer unprecedented opportunities to explore these issues, but they also introduce new challenges in data management and software implementation. Addressing these obstacles is critical for developing a more accurate and complete representation of the ToL.

This Perspective is part of a series of articles celebrating 40 years since Molecular Biology and Evolution was founded. It is accompanied by virtual issues on this topic published by Genome Biology and Evolution and Molecular Biology and Evolution, which can be found at our 40th anniversary website.

## Introduction

In 1837, Charles Darwin sketched the famous “I think” diagram on page 36 of his Notebook B. This branching depiction of evolution, now iconic, has become a cornerstone of modern evolutionary research. In *The Origin of Species*, he went as far as to suggest that every species is an endpoint of an unbroken ancestor-descendant stream of inheritance with modifications linking all organisms, forming a vast Tree of Life (ToL). This imagery has inspired biologists ever since, but until the advent of nucleotide and amino acid sequencing, the ToL could not be effectively investigated (Woese and Fox 1977). To celebrate the 40th anniversary of SMBE, this perspective explores the challenges of reconstructing the evolutionary relationships of life from the standpoint of computational and theoretical phylogenetics.

It is now clear that the entire history of cellular life is far more complex than a simple branching tree. The genome of every organism contains information derived from processes other than vertical transmission (Sagan 1967; Blais and Archibald 2021). Although this phenomenon was initially considered unique to prokaryotes, it is now widely accepted that parts of eukaryotic genomes also originated through horizontal gene transfer (HGT) (Husnik and McCutcheon 2018; Keeling 2024). Furthermore, endosymbiotic events have been pervasive throughout evolution (Sagan 1967; Bennett et al. 2024). These nonvertical transfers and mergers of genetic information make it impossible to represent life's phylogeny as a fully bifurcating tree. In fact, the ToL may reflect a small fraction of the genome (Dagan and Martin 2006) or serve as a statistical representation of the evolutionary history (O’Malley and Koonin 2011).

Therefore, one of the foremost challenges in constructing the ToL is perhaps determining how to accurately depict the genealogy of all organisms (Corel et al. 2016). This conceptual problem has been actively discussed for years (Doolittle 1999; Blais and Archibald 2021). It has been further complicated by the ample recognition that the history of genomic segments might not match the diversification of species (Maddison 1997). Additionally, because the ToL depicts only the history of cellular life, the issue of how to relate this representation with the origin and evolution of viruses and other acellular replicators has attracted attention. Although the evolutionary origins of the viral distinctive replication machinery remain unclear (Krupovic et al. 2019), many viral genes are evolutionarily related to genes found in cellular life (Koonin et al. 2015). Therefore, although arguments were made to exclude viruses from the ToL (Moreira and López-García 2009), links between the ToL and the virosphere can be established.

**Fig. 1.**
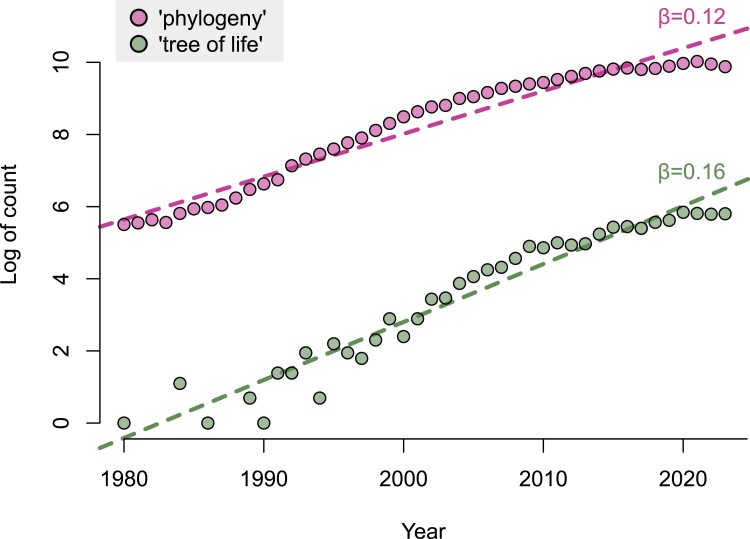
Comparative counts of the number of studies mentioning “phylogeny” and “ToL” retrieved from the Scopus database for the period 1980-2023. Both linear regression models have *R*^2^ > 0.95 and *P* < 0.001.

**Fig. 2.**
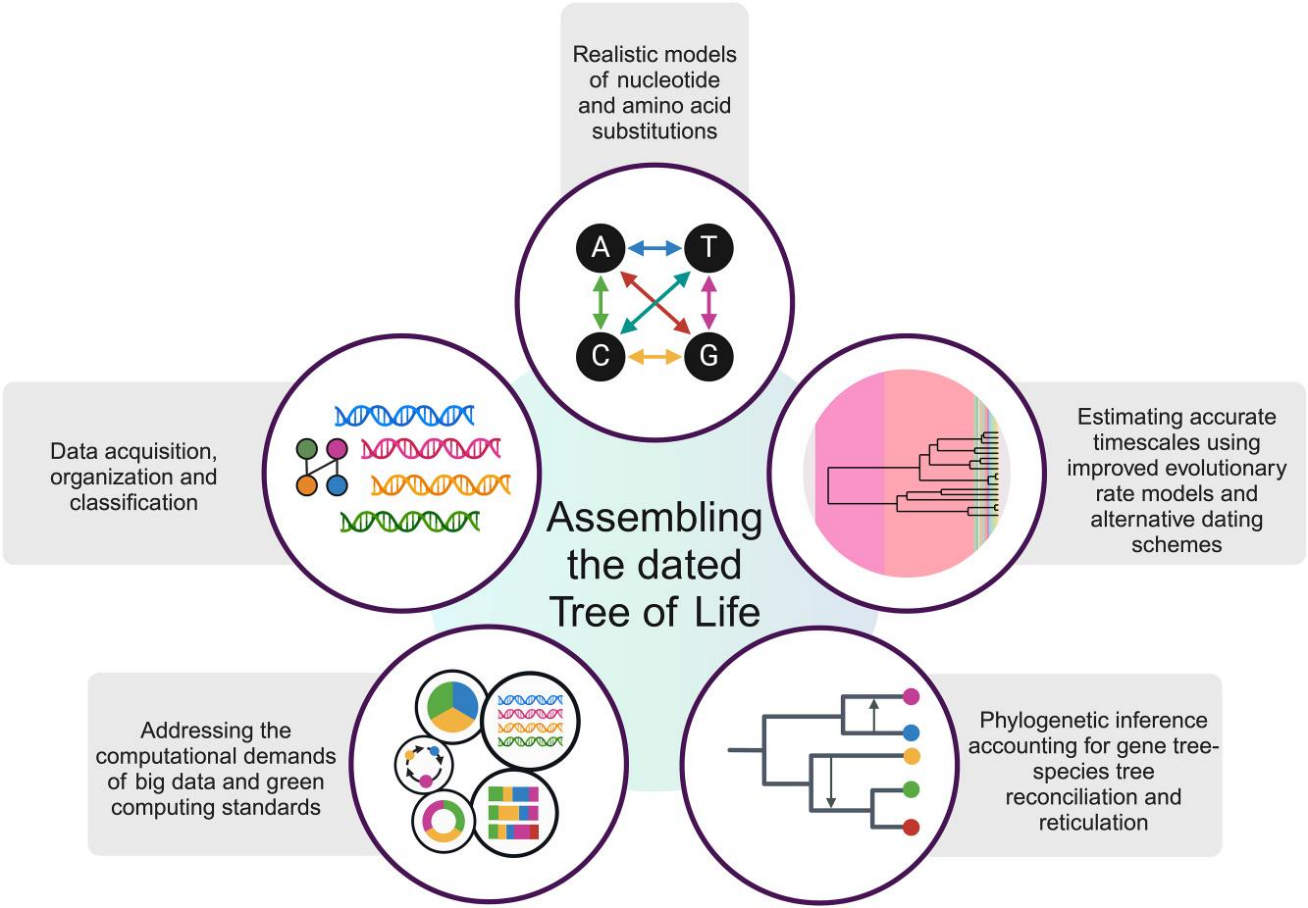
Major challenges for assembling the dated ToL.

Fueled by the increasing affordability of genome sequencing, our understanding of deep phylogenetic relationships has expanded significantly in recent decades (Spang et al. 2022; Eme and Tamarit 2024). The growing significance of the topic is underscored by the number of published papers referencing the ToL, which is increasing at a faster rate than those mentioning phylogeny broadly (Fig. 1). Here, we outline key methodological challenges biologists face in assembling the ToL, including (i) the various components of data processing; (ii) phylogenetic inference, including reconciling gene tree-species tree discordance and accounting for reticulations; (iii) the realistic modeling of nucleotide and amino acid substitutions; (iv) inferring accurate timescales and devising models of rate evolution on lineages; and (v) addressing the computational demands posed by big data (Fig. 2).

### Gathering and Organizing Sequence Data

The rapid pace of genome and metagenome sequencing is unprecedented and continues to accelerate. As more sequences become available, the critical task of filtering, classifying, and organizing this overwhelming volume of raw data cannot be overlooked (Stephens et al. 2015). Several databases have been developed to facilitate data assembly for phylogenetic studies, focusing on both unicellular and multicellular organisms (e.g. Goodstein et al. 2012; Uchiyama et al. 2019; Harrison et al. 2024). These databases, which include curated datasets and advanced phylogenomic pipelines, have allowed researchers to explore the ToL in greater depth, offering valuable resources for comparative studies (Cerón-Romero et al. 2019; Hernández-Plaza et al. 2023).

To investigate life's diversification, the standard approach involves compiling data sets of single-copy orthologs (SCOs), which are presumed to be predominantly vertically transmitted. This strategy, however, significantly limits the number of genes used, narrowing it to ∼50 when considering all living organisms (Moody et al. 2024). As we move from the last universal common ancestor (LUCA) toward the leaves of the ToL, the amount of orthologous sequence data employed to estimate its various subtrees increases, generating an imbalance. Even when studies focus on the same taxonomic level, besides sampling distinct terminal taxa, the genes used often differ (Petitjean et al. 2014; Zhu et al. 2019). Inferring SCOs for lineages separated by more than 3 billion years is difficult. Even if these genes are reliably identified, determining site-wise homology in deeply diverged sequences through alignment algorithms is far from straightforward (Landan and Graur 2009).

Because alignments are themselves estimates—a fact often neglected—phylogenetic methods have been developed to infer both alignments and trees simultaneously (Suchard and Redelings 2006) or even allow for alignment-free inference (Balaban et al. 2022). However, the effectiveness of these methods in improving the reconstruction of the ToL remains uncertain. After aligning sequences, a common step in phylogenomic pipelines is trimming positions with low phylogenetic information (Capella-Gutiérrez et al. 2009; Steenwyk et al. 2020), although this practice has been shown to reduce phylogenetic accuracy in some cases (Tan et al. 2015). For amino acid sequences, when alignments are unreliable due to high sequence dissimilarity, the structural conformation of proteins may provide evolutionary information for phylogenetic analysis (Bujnicki 2000; Malik et al. 2020). Data processing and classification algorithms are expected to continue improving, revealing previously unknown homology associations (Pavlopoulos et al. 2023).

### From Gene Genealogies to the ToL

Aside from the challenges of identifying homologous genes, theoreticians have demonstrated that the standard approach of concatenating individual alignments into supermatrices for phylogenetic reconstruction can lead to biased tree topology estimates (Kubatko and Degnan 2007). Alternatively, topological heterogeneity along the genome, arising from the independent evolutionary histories of unlinked genomic segments (“gene trees”), provides valuable information for inferring the species tree (i.e. phylogeny). Discordance between the species tree and gene trees is due to various factors, including statistical errors caused by limited sampling or model misspecification (Delsuc et al. 2005), as well as genuine biological processes (Degnan and Rosenberg 2009). Distinguishing between these causes is not straightforward. Most advancements in theoretical and computational phylogenetics over the past two decades have focused on developing methods that accommodate the various biological factors causing this topological discordance (Edwards 2009).

The first major theoretical breakthrough in this field was the development of the multispecies coalescent (MSC) model, which extends Kingman's coalescent (Kingman 1982) from single species to gene genealogies across multiple species. The MSC provides theoretical expectations for the distribution of gene trees and phylogenetic parameters, enabling the assessment of incomplete lineage sorting (ILS) (Rannala and Yang 2003; Degnan and Salter 2005). Although this model was introduced in the 1980s (Tajima 1983; Pamilo and Nei 1988), interest in this approach only surged with the advent of sequencing technologies that allowed for the assembly of multigene datasets. Currently, phylogenetic inference using MSC has become routine. However, its implementation is computationally intensive, and full parametric inference remains prohibitive in most cases, even within the Bayesian framework using Markov chain Monte Carlo (MCMC) methods (Heled and Drummond 2010). This has already prompted, and will likely continue to drive, the development of more efficient summary methods (Mirarab et al. 2014).

**Fig. 3.**
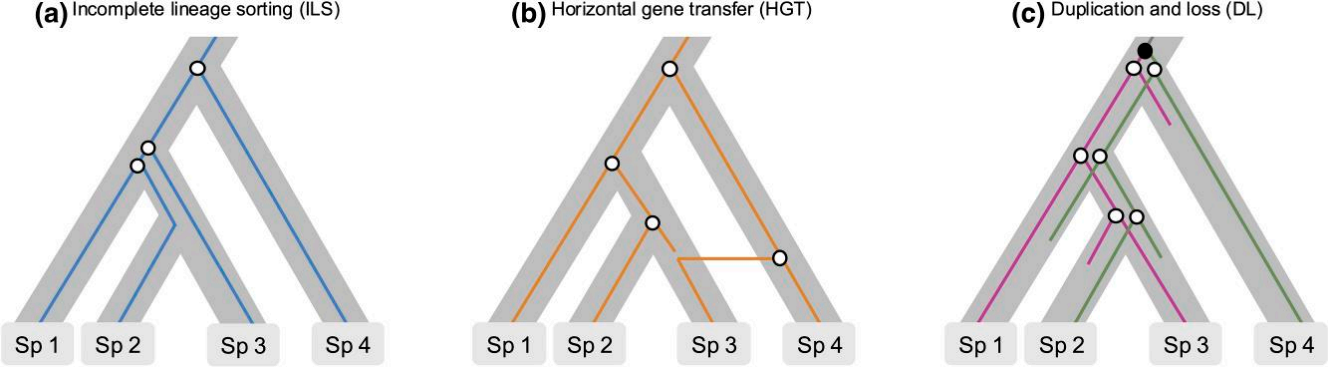
Biological processes that generate gene tree-species tree discordance. Open circles indicate coalescent events. (a) ILS can lead to discrepancies between the gene tree and the species tree; (b) HGT, followed by the fixation of the transferred allele, results in a genealogy that differs from the species tree; (c) Ancestral gene duplication, marked by a filled black circle, and subsequent differential loss of the duplicated copies contribute to discordance between the gene tree and the species phylogeny.

Although MSC accounts for ILS, it addresses only one of the causes contributing to topological mismatches between gene genealogies and the species phylogeny. Gene duplication and loss (DL), along with HGT, are other common processes generating gene-species tree discrepancy, both inducing nonorthologous relationships between sequences (Fig. 3). Therefore, to expand the number of genes used to assemble the ToL beyond SCOs, paralogy and nonvertical transmission found in gene families must be addressed. In such cases, gene trees contain nodes unrelated to between-species divergence, and these must be reconciled with the species tree using methods that either incorporate explicit models of gene family evolution accounting for DL and HGT (Szöllősi et al. 2013) or rely on parsimony (Bayzid and Warnow 2018). Recent methodological approaches use alignments of entire gene families to estimate the species phylogeny, thereby overcoming the limitations of relying exclusively on SCOs (Boussau et al. 2013; Morel et al. 2024).

Ideally, phylogenetic inference with reconciliation should incorporate the MSC process in addition to considering duplication, loss, and transfer (DLT). However, co-estimating species and gene trees using an MSC + DLT model is complex (Mirarab et al. 2021). Consequently, efforts have primarily focused on estimating phylogenies under MSC while at least considering DL (Rasmussen and Kellis 2012; Zhang et al. 2020). Recent developments model the MSC process within networks, incorporating introgression between lineages and thereby creating branches where nonvertical transfer of information has occurred (Wen et al. 2018). While introgression and HGT are different phenomena, they both involve nonvertical transmission. Thus, networks provide a theoretical foundation for integrating DLT with MSC into a comprehensive inferential framework.

Although promising, networks introduce substantial conceptual changes. Biologists are accustomed to reading the evolutionary history through acyclic graphs, making the interpretation of reticulations more difficult. Moreover, statistical evaluation of trees typically relies on branch support values that require summarizing topologies, such as the bootstrap and the Bayesian posterior probabilities. Network summarization is computationally more demanding, necessitating adjustments to these traditional metrics (Solís-Lemus et al. 2017). These difficulties pave the way for future research and methodological innovations.

Most phylogenetic algorithms produce unrooted tree topologies, leaving the temporal direction undefined. In fact, one of the most reported challenges across the ToL is determining the root of various lineages. Tree rooting is typically achieved using outgroups, but this approach becomes even more problematic when addressing the LUCA, as no living outgroups exist. In such cases, rooting can be achieved using gene families that duplicated before the divergence of all extant cellular life (Gogarten et al. 1989). However, since duplicate copies may be unavailable and establishing paralog groups is also prone to error, alternative methods can be applied (Tria et al. 2017). Finally, tree rooting can also be implemented using DLT reconciliation methods (Williams et al. 2017; Coleman et al. 2021). Moreover, recently proposed phylogenetic inference algorithms accounting for DLT yield rooted tree topologies without requiring outgroups (Morel et al. 2022, 2024). Correct root placement is crucial for resolving key questions related to the evolution of major lineages, including Eukarya (Cerón-Romero et al. 2022), Archaea (Petitjean et al. 2014), and Bacteria (Coleman et al. 2021).

### Modeling Sequence Evolution

Regardless of the phylogenetic method, tree inference from alignments relies on explicit nucleotide or amino acid substitution models. Biologically, these models must account for numerous biochemical and life-history changes that genomes have undergone over billions of years and countless rounds of DNA replication. Developing realistic models of sequence evolution is a difficult task, and it underlies several unresolved issues related to the ToL (reviewed by Williams et al. 2021). When models are misspecified, systematic errors resulting in long-branch attraction (Felsenstein 1978) and other phylogenetic anomalies might emerge. Because of the massive number of sampled sites in genomic studies, biased estimates with seemingly low uncertainty are frequently obtained.

For improving sequence evolution models, two main factors are recurrently cited: allowing for evolutionary rate heterogeneity and incorporating multiple site-specific profiles. Rate heterogeneity among sequence sites is typically addressed using a discretized Gamma distribution (Yang 1994), allowing the site likelihood to be summed over a predefined number of rate categories over the entire tree. This approach assumes that, while among-site variation exists, site-specific rates are homogeneous across the tree branches (Goloboff et al. 2019). For inferring the deep divergences of the ToL, this assumption may be unrealistic and should ideally be reconsidered (Tuffley and Steel 1998; Galtier 2001).

In addition to rate variation, accounting for different substitution profiles and compositional heterogeneity among sites also improves the accuracy of phylogenetic relationships between species that diverged deeper in time (Kapli et al. 2021). This can be achieved by partitioning data in advance or using mixture substitution models that integrate the likelihood function across different compositional categories (Lartillot and Philippe 2004). Unlike partition models, mixture models offer the advantage of not requiring predefined site-to-model assignments. Addressing both rate and substitution profile heterogeneity simultaneously requires more advanced models (Crotty et al. 2020). However, the benefit of greater complexity is counterbalanced by the downside of an expanded parameter space, which raises the time needed for analysis (Wang et al. 2018). Researchers are thus actively developing approximate solutions to balance accuracy and computational efficiency (e.g. Banos et al. 2024).

### Estimating the Timescale of Life

As new lineages are discovered and added to the ToL, establishing the timeline of species diversification is a key step in uncovering the major patterns of evolution (Hedges et al. 2015). To convert branch lengths, measured in units of substitutions per site, into units of absolute time, additional information on either time, substitution rates, or both is required. Temporal information is primarily derived from fossils, while substitution rates are sourced from empirical estimates (e.g. Bergeron et al. 2023) or borrowed from previous molecular dating studies. Since the early 2000s, timescale estimation has been conducted predominantly within a Bayesian framework (Kishino et al. 2001). In this approach, branch lengths are decomposed using an explicit model of how substitution rates change from branch to branch, combined with calibration information via probabilistic distributions (Dos Reis et al. 2016; Bromham et al. 2018).

Although several subtrees of the ToL have abundant fossil records for calibrating node ages, these lineages are mostly younger than 500 million years, and they are heavily biased toward multicellular organisms with hard tissues. To improve the precision of age estimates across the entire phylogeny of life, fossil calibrations from single-celled organisms older than one billion years are required. However, such findings are rare, and only a few fossils have been used for this purpose (see detailed paleontological justifications in Moody et al. 2024). One key result from recent timescales is that the age of the LUCA is approximately 4 billion years, which is close to the maximum bound set by the Moon-forming impact (Betts et al. 2018; Kumar et al. 2022; Craig et al. 2023).

Dating the ToL can be achieved using genes that originated from duplication events pre-LUCA. Although very few gene families meet this criterion, this information can be leveraged to cross-brace the nodes of the phylogeny, effectively doubling the number of calibrated nodes (Shih and Matzke 2013; Mahendrarajah et al. 2023; Moody et al. 2024). When incorporated into a model of gene family evolution, HGT can also inform divergence times, as this process occurs between lineages that coexisted in the past (Szöllosi et al. 2012; Davín et al. 2018; Wolfe and Fournier 2018). While node calibration has been the standard procedure in dating the ToL, Bayesian divergence time priors can be adjusted to account for the constraints imposed by HGT (Szöllõsi et al. 2022).

Regarding evolutionary rate variation across the ToL, Bayesian prior distributions used for modeling substitution rate evolution are generally classified as either autocorrelated or uncorrelated (reviewed by Mello and Schrago 2024). When constructing a dated ToL, it is biologically expected that rate autocorrelation between branches would diminish over more than 3 billion years of evolution. However, due to the vast time span involved, even uncorrelated models–which assume a single underlying prior distribution for substitution rates along the phylogeny–might be inadequate. As a result, studies often report node age estimates using several rate models (Betts et al. 2018; Moody et al. 2024).

Due to computational limitations, a full Bayesian inference of the dated ToL, which involves estimating both the phylogeny and timescale, is rarely implemented. The typical approach is to fix the tree topology for estimating divergence times using the MCMC algorithm. To speed up computations, approximations of the tree likelihood can be employed (Kishino et al. 2001; Reis and Yang 2011). Fixing the tree topology, however, may reduce the impact of nucleotide and amino acid substitution models on the estimates of branch lengths and, consequently, divergence times (Tao et al. 2020).

### Integrative Approaches and Fast Methods

The recent data deluge has presented many computational challenges in assembling the universal dated phylogeny of organisms. Since it is impractical to re-estimate the entire ToL each time a new genome becomes available, alternative strategies are necessary (Kramer et al. 2023). One obvious solution is to combine the results from the hundreds of independent phylogenetic analyses published each month in specialized journals, thereby parallelizing the assembly of the ToL and pooling the efforts of researchers worldwide. Such integrative approaches require acquiring, curating, and developing new analytical tools for data assembly and statistical evaluation of the resulting megaphylogeny.

Integrative data acquisition and curation have been implemented in many databases (e.g. Kumar et al. 2022), but fewer studies have addressed the best methods for combining information from multiple sources, summarizing, and potentially evaluating the various parameters of the ToL (Truszkowski et al. 2023; Sánchez Reyes et al. 2024). This is crucial because many clades of the ToL are understudied, leading to biased sampling in the published phylogenies that contribute to the construction of subtrees. Ideally, these knowledge databases should consider and weigh the factors influencing phylogenetic inference, such as sequence length, taxonomic sampling, and the confidence metrics associated with the original trees (e.g. topological support for branches and credibility intervals for divergence times). Collecting this information would be much easier if published phylogenies were required to be stored in public, free-of-charge databases in standardized, machine-readable formats, similar to how nucleotide and amino acid sequences are uploaded to repositories. In case dated phylogenies are to be estimated de novo, methods that optimize computational time are essential, and benchmarking studies comparing the relative performance of time-consuming and faster alternatives are invaluable in the genomic age (Mello et al. 2017; Barba-Montoya et al. 2021; Costa et al. 2022).

## Conclusion and Future Developments

Computational and theoretical phylogenetics has advanced significantly since the early works of the 1960s, and newer approaches are essential for estimating the deep evolutionary relationships in the ToL. Nevertheless, several fundamental questions remain unanswered: Is there sufficient empirical evidence to support the view that vertical transmission events primarily drive the evolution of life? Or might a reticulated phylogeny provide a more accurate depiction of life's deep evolutionary history? If the latter, how can we test competing hypotheses, interpret, and assign statistical support to the branches (edges) of such networks? Additionally, divergence time estimation would benefit from accounting for nonvertical events. In the widely used Bayesian framework, the adoption of priors that consider cyclic graphs has been introduced, offering promising avenues for integrating multiple sources of genetic transmission in dating the ToL (Flouri et al. 2020).

It is safe to say that the coming years will witness a series of methodological advancements that address many of these questions. As in other areas of evolutionary biology, future developments will likely be driven by the influx of high-quality genomes resulting from advances in sequencing technologies. Also in the technical domain, the impressive performance of machine learning algorithms and AI are likely to play a significant role in collecting and organizing phylogenomic data and improving pipelines (e.g, Kirilenko et al. 2023). To formulate testable hypotheses about the history of life using sequence data, biologists must tackle the management of data overflow–from storage to analysis–and the energy demands required throughout the process (Kumar 2022). This environmentally sustainable framework for conducting bioinformatic research is called green computing (Grealey et al. 2022).

These new standards should be considered by both theoreticians and software developers, as analytic resources are limited and unevenly distributed within the scientific community. Overcoming these obstacles is a crucial step toward the democratization of evolutionary science. Fully assembling the dated ToL will require substantial efforts in sequencing and analysis of unsampled species, particularly those from biodiversity hotspots primarily located in the Global South. Therefore, the active involvement of the local scientific community in this endeavor should be encouraged. In this regard, scientific societies have a central role, especially in promoting initiatives that address this issue, such as those implemented by SMBE (Eyre-Walker and Katz 2024).

## Data Availability

No new data were generated or analyzed in support of this research.
